# Antimicrobial Activities of Essential Oils of Different *Pinus* Species from Bosnia and Herzegovina

**DOI:** 10.3390/pharmaceutics16101331

**Published:** 2024-10-15

**Authors:** Snježana Mirković, Vanja Tadić, Marina T. Milenković, Dušan Ušjak, Gordana Racić, Dragica Bojović, Ana Žugić

**Affiliations:** 1 PHI Hospital “Sveti Vračevi”, Srpske vojske 53, 76300 Bijeljina, Bosnia and Herzegovina; 2 Institute of Medicinal Plants Research “Dr. Josif Pančić”, Tadeuša Košćuška 1, 11000 Belgrade, Serbia; 3 Faculty of Pharmacy, University of Belgrade, Vojvode Stepe 450, 11000 Belgrade, Serbia; 4 Institute of Molecular Genetics and Genetic Engineering, University of Belgrade, Vojvode Stepe 444a, 11042 Belgrade, Serbia; 5 Faculty of Ecological Agriculture, University Educons, Vojvode Putnika 87, 21208 Novi Sad, Serbia; 6 Faculty for Food Technology, Food Safety and Ecology, University of Donja Gorica, Oktoih 1, 20000 Podgorica, Montenegro

**Keywords:** *Pinus* species, essential oils, GC, chemical composition, antimicrobial activity, synergy, bioeconomy

## Abstract

Background/Objectives: The emergence of antimicrobial resistance has urged researchers to explore new antimicrobial agents, such as essential oils (EOs). The aim of this study was to examine chemical composition and antimicrobial activity of the EOs from the needles and green cones of four *Pinus* species (*Pinus mugo* Turra., *P. nigra* J.F., *P. syilvestris* L., and *P. halepensis* Miller) from Bosnia and Herzegovina. Methods: Chemical profiles of EOs were assessed by gas chromatography, while microdilution method was used to test their antimicrobial activity. A synergistic action of EOs and gentamicin was investigated by the checkerboard assay. Results: The chemical composition of the tested EOs showed a high percentage of α-pinene, (*E*)-caryophyllene, limonene, germacrene D, myrcene, and δ-3-carene. EO from green cones of *P. sylvestris* showed high efficiency against *S. aureus* and *E. faecalis*. The MIC of *P. nigra* cones’ EO was 100 μg/mL against *E. coli*. The EO of *P. halepensis* green cones demonstrated the strongest activity against *E. faecalis.* EOs of *P. halepensis* needles and green cones exhibited the highest activity against *C. albicans.* Further, synergistic interaction was detected in combination of the selected EOs/gentamicin toward *S. aureus* and *K. pneumoniae*. Conclusions: Among the tested EOs, oils of *P. sylvestris* cones and *P. halepensis* cones and needles showed the greatest antimicrobial activity. The same EOs and EO from *P. nigra* cones displayed synergistic potential in combination with gentamicin, supporting their utilization as antimicrobial agents alone or in combination with antibiotics, which is in line with their ethnopharmacological usage and circular bioeconomy principles.

## 1. Introduction

The genus *Pinus* (Pinaceae) comprises 250 species and is a dominant forest component in the Northern Hemisphere [1,2]. The medicinal and aromatic properties of the chemical compounds of pine (essential oil, turpentine, and resins) make it one of the most popular plants throughout all civilization [3].

The various parts of the *Pinus* species have ethnomedicinal usages as treatments for skin conditions, asthma, wounds, bronchitis, the common cold and cough, cardiac disease, muscle disorders of infectious, rheumatic or neuralgic origins, etc. [4,5]. In the ethnobotany of Bosnia and Herzegovina, the original medicinal Bosnian “*mehlemi*” (ointments) are known, and they were made from the resin of *Abies* and *Pinus* species and fresh parts of plants. *Pinus mugo* Turra, *P. nigra* J.F., *P. sylvestris* L., and endemic *P. heldreichii* Christ have been used for the treatment of different skin conditions and wound healing [6,7].

*Pinus* species are reported to have various biological effects, such as antioxidative, anti-inflammatory, antimicrobial, antimutagenic, and anticancer activities assessed in vitro [8,9,10]. These medicinal plants have been reported to have cardiovascular benefits and stimulate both cellular and humoral immune responses. Some species are frequently utilized to treat various health-related conditions, such as wound healing, pulmonary, urinary, hepatic, and respiratory diseases [11,12,13,14,15]. Pine oils are widely used as odors in the soap and perfume manufacturing industries [16].

Antimicrobial resistance has emerged as the biggest challenge, which threatens the health of society [17]. As antibiotics became more widely used, bacteria responded by developing various forms of resistance to these treatments, which has rapidly accelerated, thus creating a serious and global problem [18]. The development of drug combinations has shed light on a novel approach in controlling resistant pathogens [19]. Essential oils (EOs) have been found to act as synergistic enhancers in this regard. Namely, they may not produce any significant inhibitory effects when used alone, but when they are used in combination with standard drugs, the combinatory effect surpasses their individual performance and produces enhanced antimicrobial activity [20].

EOs are a very interesting group of secondary metabolites that are potentially useful sources of antimicrobial compounds [21]. In association with antibiotics, EOs reduce adverse effects and the minimum effective dose of antibiotics in the treatment of infections. Most importantly, these synergistic combinations targeting resistant bacteria may have novel and multiple mechanisms of action that could overcome microbial resistance [22]. EOs of *Pinus* sp. have already been studied for antimicrobial properties against bacteria and fungi proving that they may serve as a source of antimicrobial agents [3,23,24,25,26,27]. However, very little is known about the biological activities of *Pinus* sp. EOs from Bosnia and Herzegovina. The chemical composition and properties of the EOs are greatly influenced by factors such as the environmental conditions (place where the plant is grown, the soil, the air temperature during collection, climate, collection time), genetics, sampling techniques, EO extraction methods, chromatographic processing, etc. As a result, EOs obtained in different countries may show different antimicrobial effects [28]. In addition, information describing the pharmacological activity of EOs from green cones is very limited in the literature.

Chen et al. [29] reported that forest areas cover approximately 43% of land in Europe, implicating that its biomass covers 89.3% of total biomass amount. Bioactive compounds of pine species’ EOs are recognized as lucrative resources which are economically viable, particularly in the food and medicine sectors. However, needles and green cones of pine species are valuable biomass resources in the agroforest industry, estimating that pine needles are 30% of total pine tree mass [30]. High-quality pine EOs, being explored for their beneficial traits due to high antioxidant and antimicrobial effects as components of biopesticides in ecological agriculture application, provide economically valuable forest products. However, the waste of pine needles as the source of these EOs presents a threat to forest fires caused by their high flammability [29,31,32]. Eco-friendly approaches, which gather the production, use, and transformation of bioresources, are encompassed in the concept of the bioeconomy. Establishing pine biomass to bioenergy processes is one of the directions that follow sustainable development goals (SDGs), integrating environmental, social and economic aspects of sustainable food needs and at the same time ensuring the preservation of forest resources [33]. Estimating the nutritional composition of pine needles and cones, the main percentage goes to cellulose/hemicellulose, ranging from 57% in cones and 68% in needles, and is followed by approximately 30–40% of lignin, extractives, ashes, and minerals. The composition percentage of needles mainly varies on genotypic, ecological, and seasonal factors [34]. Relatively high lignin contents make pine biomass prosperous in sustainable energy aspects, as it can be used in the form of condensed briquettes or pellets, producing high-volume energy [35]. Biochar production from pine needles has gained great attention in the energy sector, due to high-heating volume [36]. The ability to absorb xenobiotic dyes in wastewater treatments highlight pine needles to be used in bioremediation processes. Bio-composites made of biodegradable plastic showed increased tensile strength reinforced with *P. roxburghii* needles [37]. Due to the high content of lignocellulose in pine needles, application for producing bio-ethanol is an economically friendly alternative to fossil fuels [38].

Therefore, the aim of the present study was to investigate the in vitro antimicrobial activity of the EOs isolated from the fresh needles and cones of *Pinus* sp. (*P. mugo* Turra, *P. nigra* J.F., *P. sylvestris* L., *P. halepensis* Miller) against diverse and clinically relevant bacteria and one strain of yeast, as related to their chemical composition. Interactions between EOs and conventional antibiotics (gentamicin) were investigated to determine if synergistic interactions might occur.

## 2. Materials and Methods

### 2.1. Plant Material

The needles and green cones of four pine species (*P. mugo* Turra., *P. nigra* J.F., *P. syilvestris* L., and *P. halepensis* Miller) were collected between July and August 2020, from the area of Čvrsnica mountain and Neum (Bosnia and Herzegovina). Plant identity was verified and herbarium voucher specimens were deposited at the Institute for Medicinal Plant Research “Dr. Josif Pancic”, Belgrade. Before EOs’ isolation, plant material was stored at −24 °C.

### 2.2. Microorganisms

Laboratory control strains of microorganisms were used for in vitro testing of antimicrobial activity of EOs. The standard strains of Gram-positive and Gram-negative bacteria: *Staphylococcus aureus* (ATCC 6538), *Enterococcus faecalis* (ATCC 29212), *Kocuria rhizophila* (ATCC 9341), *Bacillus subtilis* (ATCC 6633), *Escherichia coli* (ATCC 8739), *Klebsiella pneumoniae* (NCIMB 9111), *Salmonella* Typhimurium (ATCC 14028), *Pseudomonas aeruginosa* (ATCC 9027), *Acinetobacter baumannii* (ATCC 19606), and one strain of yeast *Candida albicans* (ATCC 10231) were used. Müller–Hinton agar was used for cultivation and maintenance of bacteria and Sabouraud dextrose agar for *C. albicans.*

### 2.3. Isolation of Essential Oils

The fresh needles and cones of each species were cut into small pieces and separately subjected to hydrodistillation using a Clevenger-type apparatus (Medilab, Ambala Cantt, Haryana, India) for 2 h [39]. The obtained EO was extracted with diethyl ether and dried over anhydrous sodium sulfate. After filtration, the solvent was removed under a gentle stream of nitrogen at room temperature in order to exclude any loss of the EO.

### 2.4. Chemical Analysis of Essential Oils

Quantitative and qualitative data of EOs were obtained by gas chromatography (GC) and gas chromatography–mass spectrometry (GC–MS) analyses.

#### 2.4.1. Gas Chromatography—GC

Gas chromatography analysis of EOs was carried out on an HP-5890 Series II GC apparatus (Hewlett-Packard, Waldbronn, Germany), equipped with split-splitless injector and automatic liquid sampler, attached to HP-5 column (25 m × 0.32 mm, 0.52 μm film thickness) and fitted to flame ionization detector (FID). Carrier gas flow rate (H_2_) was 1 mL/min, split ratio 1:30, injector temperature was 250 °C, detector temperature 300 °C, while column temperature was linearly programmed from 40 °C to 260 °C (at rate of 4 °C/min), and then kept isothermally at 260 °C for 10 min. Solutions of samples dissolved in chloroform-MeOH mixture were consecutively injected in an amount of 1 μL. Area percent reports, obtained as a result of standard processing of chromatograms, were used as the basis for the quantification analysis. The percentage composition of the EOs was computed from GC peak areas with the response factor considered to be 1.

#### 2.4.2. Gas Chromatography–Mass Spectrometry (GC–MS)

The same analytical conditions as those mentioned for GC-FID were employed for GC–MS analysis, along with column HP-5MS (30 m × 0.25 mm, 0.25 μm film thickness), using HP G 1800C Series II GCD system (Hewlett-Packard, Palo Alto, CA, USA). Helium was used as a carrier gas. Transfer line was heated at 260 °C. Mass spectra were acquired in EI mode (70 eV); in *m*/*z* range 40–450. The amount of 0.2 μL of sample solution in chloroform:MeOH mixture was injected. The components of the extracts were identified by comparison of their spectra to those from Wiley 275 and NIST/NBS libraries using different search engines. Calibration was performed using linear n-paraffins mixture (C6-C40) as a standard. The experimental values for retention indices were determined by the use of calibrated Automated Mass Spectral Deconvolution and Identification System Software (AMDIS ver. 2.1), compared to those from the available literature, and used as an additional tool to confirm the MS findings.

#### 2.4.3. Antimicrobial Activity

Minimum inhibitory concentrations (MICs) of EOs were determined by the broth microdilution method according to the Clinical and Laboratory Standards Institute guidelines [40]. Tests were performed in Müller–Hinton broth (MHB) for the bacterial strains, and in Sabouraud dextrose broth for *C. albicans*. Overnight broth cultures were prepared for each strain, and the final concentration in each well was adjusted to approx. 10^6^ or 10^7^ CFU/mL for bacteria and yeast, respectively. The EOs were dissolved in 1% dimethylsulfoxide and diluted to the desired concentrations using Müller–Hinton broth. Previous studies investigated antimicrobial properties of DMSO at concentrations ranging from 0 to 20% against various microorganisms. Importantly, only at levels above 5% DMSO, bacteriostatic activity was detected [41]. After incubation for 24 h at 35 °C in aerobic conditions, MICs were determined. All of the MIC determinations were performed in duplicate and two positive growth controls were included. MIC values were determined as the lowest concentrations of EO or antibiotic that inhibited visible growth of microorganisms. Each broth microdilution test was repeated three times. EOs with MIC values lower than 100 μg/mL, between 100 and 500 μg/mL, and between 500 and 1000 μg/mL, were considered to be promising, moderately active, and weak antimicrobials, respectively. Samples with MIC values greater than 1000 μg/mL were deemed inactive [42].

#### 2.4.4. Evaluation of Synergistic Effect

Checkerboard method was used to evaluate combined effects of the EOs and antibiotics (gentamicin) and to determine type of interactions (synergistic, additive, indifferent, or antagonistic). In brief, the method was performed in 96-well polystyrene microtiter plates, by pouring decreasing concentrations of tested EOs and two-fold dilutions of examined antibiotics, lower than previously determined MICs. The EOs were prepared as described above and diluted using the Müller–Hinton broth to tested concentrations. Each well was filled with the same amounts of tested agents (50 μL) and 100 μL of bacterial suspension. The bacterial suspension was prepared as described above. After the incubation of plates for 18–24 h at 35 °C, MICs were determined as the lowest concentrations of combinations, where visible growth was absent. Types of interactions were determined by calculating the fractional inhibitory concentration index (FICI) values using the following formula:

FIC index (FICI) = FIC_A_+ FIC_B_ (1)

(2) $${\mathrm{FIC}}_{A}=\frac{MIC\;of\;\left(A\right)\;in\;combination}{MIC\;of\;\left(A\right)alone}$$

(3) $${\mathrm{FIC}}_{B}=\frac{MIC\;of\;\left(B\right)in\;combination}{MIC\;of\;\left(B\right)alone}$$

where (A) is EO and (B) is antibiotic.

The FICI values were interpreted as a synergistic effect when FICI ≤ 0.5; an additive effect when 0.5 < FICI < 1; an indifferent effect when 1 < FICI < 4; and an antagonistic effect when FICI > 4 [43].

## 3. Results

As seen in Table 1, the chemical composition of the tested EOs revealed all tested EOs to be most abundant in monoterpene hydrocarbons, followed by sesquiterpene hydrocarbons, with the exception of EO isolated from *P. nigra* cones (PNC), where monoterpene hydrocarbons in the highest amount (44.14%) were followed by oxygenated diterpenes (23.55%). A high percentage of α-pinene in EOs was found in *P. nigra* needles (PNN) (54.42%), *P. halepensis* cones (PHC) (47.47%), *P. nigra* cones (PNC) (40.00%), *P. sylvestris* needles (PSN) (39.82%), *P. sylvestris* cones (PSC) (37.86%), *P. halepensis* needles (PHN) (17.02%), and *P. mugo* needles (PMN) (11.18%). (*E*)-Caryophyllene was found in high percentage in EOs in *P. halepensis* needles (PHN), *P. mugo* cones (PMC), *P. nigra* cones (PNC), *P. halepensis* cones (PHC), and *P. sylvestris* cones (PSC) (24.69%, 21.07%, 14.00%, 11.70%, and 9.13%, respectively). (*11E,13Z*)-labdadien-8-ol appeared only in the EO of *P. nigra* cones (PNC) (18.83%). Germacen D was one of the main compounds of *P. nigra* needles (PNN) (16.34%) and *P. mugo* cones (PMC) (16.30%). Myrcen was found in high percentage in the EOs of *P. halepensis* needles PHN, *P. halepensis* cones PHC, and *P. sylvestris* cones PSC (24.65%, 14.61%, and 13.78%, respectively). EOs in *P. mugo* cones and needles (PMC and PMN) contained a high amount of δ-3-carene (23.36% and 19.95%, respectively) (Table 1).

Antimicrobial activity of the EOs was tested against nine bacterial strains and *C. albicans* by using the broth microdilution method. Determined MIC values are presented in Table 2.

As seen in Table 2, tested EOs, isolated from *Pinus* species, were shown to possess inhibitory action against tested isolates in the range of 100–1000 μg/mL (MICs). Among the tested oils, EOs isolated from needles and cones of *P*. *halepensis* (PHN, PHC), and cones of *P. sylvestris* (PSC) exhibited the best overall antimicrobial action, while EOs from needles of *P. sylvestris* (PSN) and needles and cones of *P. mugo* (PMN and PMC) and *P. nigra* (PNN and PNC) showed weaker antimicrobial potential against tested microorganisms, with the exception of PNC that was surprisingly the most active sample of all tested EOs against *E. coli.*

All samples were inactive against *A. baumannii*. Similarly, most samples were inactive against *P. aeruginosa*, with the exception of EOs from PSC and PHN revealing MICs of 1000 and 800 μg/mL, respectively (Table 2). The most tested EOs revealed weak activity against *S. typhimurium*, except EO from PHC that exhibited moderate activity with a MIC of 200 μg/mL. EOs from PMN, PMC, and PHN showed moderate activity against *K. rhizophila*, while the rest of the EOs were less active (Table 2). MIC values of EOs from PNN, PSC, PHN, and PHC revealed moderate activity against *K. pneumoniae*. Most of the tested EOs were inactive against *B. subtilis*, except PMN with weak, and PHN and PHC with moderate activity (Table 2). Alike, most EOs showed weak activity against *E. faecalis*, while PSC and PHN exhibited high efficiency against this bacterium (100 μg/mL) and PHN followed with a MIC of 150 μg/mL. Similar findings were observed for *S. aureus*, where PSC demonstrated high activity (100 μg/mL), tailed by PHN and PHC, with a MIC of 150 μg/mL. Overall, *E. coli* and *C. albicans* were the most susceptible to the investigated EOs. As stated earlier, EO from PNC displayed the highest activity (MIC was 100 μg/mL), EO from PNN was the least active (MIC was 600 μg/mL), while the rest of the EOs revealed moderate activity (Table 2). Regarding activity against *C. albicans*, the most active EOs were from PHN and PHC (MICs were 100 μg/mL), the least active was EO from PMN, while the rest of the EOs showed moderate activity (Table 2).

To evaluate potential synergism between EOs and antibiotics, antimicrobial activity of different combinations of the latter agents was tested against three bacterial species (*S. aureus*, *E. coli*, and *K. pneumoniae*), selected based on their clinical significance and previously obtained MICs. Determined interactions between gentamicin and the EOs of *Pinus* sp. PSC, PNC, PHC, PHN, which previously revealed the best antimicrobial activity, are presented in Table 3. FICI values of the investigated EOs ranged from 0.2875 to >1. Synergy (FICI ≤ 0.5) was detected in combinations of all EOs and gentamicin against *S. aureus* and *K. pneumoniae*, the additive effect (FICI 0.5–1) was registered in the combination of EOs from PHN, i.e., PHC against *E. coli*. The EOs of PNC and PSC, exerted indifferent effect (FICI > 1) against *E. coli*, when combined with gentamicin. The results regarding synergy against *S. aureus* and *K. pneumoniae* suggest the possibility to inhibit bacterial growth by applying a combination of gentamicin and EOs at concentrations decreased by 4–8-fold and 4–26.6-fold, respectively (Table 3), when compared to the obtained MIC values of the latter agents.

## 4. Discussion

EOs are composed of numerous different chemical compounds, and their antimicrobial activity might be attributed to changes in the chemical components [44]. The chemistry and the biological effects of the EOs isolated from different pine species have been intensively studied, particularly in the pine needles [1,4,27,45,46,47,48]. On the other hand, there are a few studies that refer to the chemical composition of the EOs isolated from cones [49,50,51].

In our study, the chemical composition of the EOs from *Pinus* sp. was different depending on the investigated species, as well as the part of the plant (Table 1). The major compounds of the PMN and PMC were δ-3-carene (23.6% and 19.95%, respectively), (*E*)-caryophyllene (5.91% and 21.07%, respectively), and germacrene D (5.59 and 16.30%, respectively). Comparing our results to previously reported data, the chemical profile was similar to that observed in *P. mugo* EOs originating from the Kosovo area [52]. On the other hand, *P. mugo* EOs from North Macedonia, Greece, and Serbia, [53,54,55] lacked (*E*)-caryophyllene and germacrene D, but contained α-pinene, β-phellandrene, and α-terpinolene, detected in our samples (PMN and PMC) as well. In contrast to the mentioned studies where the EO contained α-pinene as the principal component, in our study, the presence of this terpene was found only in small amounts (3.39% and 1.89% in EO from PMC and PMN, respectively). The different origins of the collected plants might explain these disagreements [52,53,54].

In the present study, the dominant compound in the EOs of the PNC and PNN was α-pinene (40.00% and 54.42% respectively). Aside from α-pinene, dominant compounds in EO from PNC were (*11E,13Z*)-labdadien-8-ol and (*E*)-caryophyllene (18.83 and 14.00%, respectively), while in EO from PNN, the main compounds were germacrene D and (*E*)-caryophyllene (16.34% and 8.50%, respectively) [45,56,57]. Comparing our results to previously reported chemical composition of *P. nigra* EOs, the main difference was in detected diterpenoid (*11E,13Z*)-labdadien-8-ol in PNC, as well as in the presence of manool oxide in significantly lower percentage [27,57,58].

α-Pinene was identified as the main compound in the EOs of PSC and PSN as well (37.86% and 39.82%, respectively). The concentration of myrcene in PSC was 13.78%, followed by (*E*)-caryophyllene (9.13%). Aside from α-pinene detected in PSN, none of the identified components exceeded 10%, while the main constituent of EO from PSN reported in previous studies [45,46] was δ-3-carene, followed by α-pinene, δ-cadinene, β-pinene, and camphene.

In the EOs of PHC and PHN, the major compounds were found to be α-pinene (47.47% and 17.02%, respectively), (*E*)-caryophyllene (11.70% and 24.69%, respectively), and myrcene (14.61%, and 24.65%, respectively). β-caryophyllene (40.31%) was identified as the main compound in the EOs in cones of *P. halepensis* collected in Algeria, followed by α-humulene (7.92%) and aromadendrene (7.1%) [57]. Compared to our results, a similar chemical profile was reported for the needle EO of *P. halepensis* from West Northern of Algeria [3] and various Tunisian regions [59], admittedly with different percentages in the latter. In contrast, Aloui et al. reported α-pinene as the major compound of needle EO from *P. halepensis* [60], unlike our results emphasizing (*E*)-caryophyllene and myrcene as main constituents.

Previous studies on the antimicrobial activity of *Pinus* species from Bosnia and Herzegovina are very scarce, especially when it comes to EOs of green cones, as well as the synergism of EOs with antibiotics. Up to now, there are no available reports on the biological activities of EOs isolated from the green cones of tested *Pinus* species. Additionally, only a few studies on the antimicrobial activity of green cones’ EO of some other pine species (*P. brutia* and *P. koraiensis*) could be found in the literature [61,62].

The published data survey revealed that needle EOs of different *Pinus* species showed no activity against *E. coli* and *E. faecalis* [4], i.e., weak inhibitory action against pathogenic bacterial strains *K. pneumoniae*, *E. coli*, *S. aureus*, with MICs in the range of 0.62–20.00 mg/mL, that were substantially higher compared to MIC values determined in this study [63].

For instance, most of the tested EOs in our study displayed MIC in the range of 100–600 μg/mL against *E. coli*, with PNC showing the most potent antimicrobial effect. These findings support the potential application of tested EOs in helping combat health impairments, such as gastroenteritis, urinary tract infections, and systemic infections in humans and animals caused by this bacterium, additionally taking into account emerging resistance to common antibiotics [64].

Moderate antimicrobial activity of most tested pine EOs (400–800 mg/mL) was detected against *K. pneumoniae*, which causes a wide range of diseases including nosocomial pneumonia, urinary tract infections, diarrhea, and intra-abdominal infections [65]. Our finding supported the traditional usage of different parts of *Pinus* species in the treatment of respiratory problems in folk medicine [5,66,67,68].

EOs from PSC, PHN, and PHC revealed high antimicrobial activity against *S. aureus* (100–150 mg/mL), being the leading cause of skin and soft-tissue infections such as abscesses, furuncles, impetigo, and cellulitis [69]. Furthermore, it is one of the most common pathogenic bacteria isolated from wounds, in addition to *E. coli* and *K. pneumoniae* [70]. Bearing in mind the results of our study, tested pine EOs may be applicable for the treatment of wounds, which is in line with their ethnopharmacological usage [12,71,72].

*K. rhizophila*, typically considered a commensal microorganism, is being increasingly recognized as an emerging opportunistic pathogen, causing different types of infections, mostly in immunocompromised hosts with serious underlying conditions and metabolically disordered individuals [73,74]. Considering its relatively small genome size, it is surprising that each *K*. *rhizophila* strain is highly adapted to its ecological niche and capable of growing robustly in various conditions [75]. To date, no antimicrobial activity of *Pinus* species against this bacterium has been reported in the literature. In the present study, moderate activity (400–600 mg/mL) against this bacterium was reported for EOs from PMC, PMN, and PHN, while other EOs displayed weak activity toward *K. rhizophila*.

*Candida* is one of the most common human fungal pathogens that represents the most important cause of opportunistic mycoses [76]. The widespread use of antifungal drugs, particularly, has led to the development of drug resistance in the treatment of *C. albicans* infections, a problem of growing importance. This necessitates either the development of novel antifungal drugs or improved therapeutic strategy to overcome drug resistance problems by *C. albicans* [77]. EOs from needles/cones of the investigated *Pinus* species have generally shown good anti-*Candida* activity, especially from PHN and PNC (MIC = 100 μg/mL).

Overall, the demonstrated antimicrobial activity in our study justified the use of EOs from *Pinus* sp. as antiseptics for ethnotherapeutic purposes. *Pinus* species are traditionally used as antiseptics in both respiratory and urinary tract complaints, and in dermatological diseases (acne, fungal diseases, dermatologic lesions) [53,63,78,79].

Obtained antimicrobial activity of tested EOs of *Pinus* species can be related to the dominant presence of α-pinene, which was determined to be the active antimicrobial compound in EOs of *Pinus* sp. in previous studies [53,65,80,81]. It has been shown that α-pinene destroyed cellular integrity, inhibited respiration and ion transport processes, and increased membrane permeability [82]. The fact that EO of PNN (α-pinene—54.42%) exhibited lower activity in comparison to other *Pinus* sp. (α-pinene present in the range of 11.30–40%) highlighted that a higher amount of dominant antimicrobial compound did not necessarily mean higher antimicrobial potential, especially in chemically complex oils [51]. In addition to α-pinene, EOs of *Pinus* sp. investigated in this work contained limonene, caryophyllene, and myrcene, as major compounds, previously reported to display antimicrobial activity against important pathogens [4,16,50].

There are several studies examining the synergism between the EOs of *Pinus* sp. and antibiotics, where the results are incomparable to ours, because there are differences that are reflected in the species of the genus *Pinus*, the type of sample that has antimicrobial activity (EO vs. resin), or the type of microorganisms tested [79,83]. Silva et al. [83] evaluated the antibacterial potential of *P. elliottii* and *P. tropicalis* resins as well as of the diterpene dehydroabietic acid (DHA) against cariogenic bacteria. They showed the biofilm inhibition ability, as well as the synergistic effect of chlorhexidine and resins. Neither additive nor synergistic effects emerged for the combinations of one of the resins with chlorhexidine. In the study performed by Scalas et al. [79], the EO of *P. sylvestris* and α-pinene displayed good inhibitory activities against *C. neoformans*. In addition, the combination of itraconazole with the EO of *P. sylvestris* showed a good synergistic action against *C. neoformans*. In the present study, EOs of PNC, PSC, PHC, and PHN showed synergism with gentamicin against *S. aureus* and *K. pneumoniae*.

Results presented in this paper, which supported pine EOs’ antimicrobial properties, were comprehensively aligned with pine species residue applications, reutilization, and future perspectives to obtain high industrial interests [84]. A holistic approach to clean environments and improved livelihoods, aligned with SDGs and circular bioeconomy principles, offers a promising path for diverse stakeholders, such as policymakers, citizens, researchers, and industry members. By enhancing resource efficiency, minimizing waste, and creating a clean environmental system in forest areas, we can foster innovation, responsible consumption, and mitigate the effects of climate change.

## 5. Conclusions

This paper presents the first report on the antimicrobial properties of the needle and cone EOs of *P. mugo*, *P. nigra*, *P. sylvestris*, and *P. halepensis* from Bosnia and Herzegovina and the synergism between the antimicrobial activities of the investigated EOs and antibiotics. Also, in the present study, the chemical composition of the EOs isolated from needles/green cones of the investigated *Pinus* sp. was examined. In particular, α–pinene, (*E*)-caryophyllene, germacrene D, limonene, and δ-3-carene as the dominant constituents, were the most abundant compound class of the EOs of the investigated *Pinus* sp. Among the tested EOs, oils of *P. sylvestris* cones, *P. halepensis* cones, and *P. halepensis* needles showed the greatest antimicrobial activity. The difference in observed activity was mainly related to the different concentrations of pinenes in the EOs of different species, although synergistic effects with other oil compounds cannot be ruled out. According to the obtained results, the EOs of *P. nigra* cones, *P. sylvestris* cones, *P. halepensis* cones, and *P. halepensis* needles possessed synergistic potential in combination with gentamicin against *S. aureus* and *K. pneumoniae*. Based on the present results, it could be hypothesized that the antimicrobial activity and the synergistic effect of *Pinus* EOs and gentamicin were associated with the high percentage of monoterpene and sesquiterpene hydrocarbons. The results point to a high potential and completely justify the utilization of the pine EOs because of the wide antimicrobial spectrum of some investigated *Pinus* species’ EOs. Combinations of EOs and antibiotics reduced the minimum effective dose of the antibiotics and consequently, might minimize their adverse side effects and could lead to new options for the treatment of infectious diseases and emerging drug resistance. In addition, the presented findings support the usage of pine species residues as the source of antimicrobial agents, being in line with SDGs and circular bioeconomy principles.

## Figures and Tables

**Table 1:** Chemical composition of the EOs isolated from needles and green cones of *Pinus* sp.

Chemical Compounds	Kovats Index	CAS	PNC	PNN	PMC	PMN	PHC	PHN	PSC	PSN
Approximate			Percentage (%)							
Tricyclene	921	508-32-7		0.21	t	0.48	0.07	0.08	t	0.66
α-Thujene	924	2867-05-2		0.30	0.09	1.62				0.35
α–Pinene	932	80–-56-8	40.00	54.42	3.93	1.89	47.47	17.02	37.86	39.82
Camphene	946	79-92-5	0.69	1.16	1.21	1.41	0.65	0.18	0.74	3.08
Thuja-2,4(10)-diene	953	36262-09-6							0.03	
Verbenene	961	4080-46-0		0.24						
Sabinene	969	3387-41-5			0.66	1.69		2.49		
3,7,7-trimethylcyclohepta-1,3,5-triene	970	3479-89-8								
β–Pinene	974	127-91-3	2.49	3.59	2.21	6.56	3.85	3.44	6.78	6.02
Myrcene	988	123-35-3		1.22	1.38	2.41	14.61	24.65	13.78	1.69
α-Phellandrene	1002	99-83-2		0.03	0.12	0.19				0.04
*iso*-Sylvestrene	1007	1461-27-4			0.08					7.85
δ-3-Carene	1008	13466-78-9		0.02	19.95	23.36	5.10		0.07	
α-Terpinene	1014	99-86-5		0.02						0.02
*p*-Cymene	1020	99-87-6		0.05	0.31	0.19	0.09			0.22
Limonene	1024	138-86-3	0.96	2.57			5.98		6.92	
β-Phellandrene	1025	555-10-2			7.33	5.75		2.81		2.23
(*Z*)-β-Ocimene	1032	3338-55-4								0.04
(*E*)-β-Ocimene	1044	3779-61-1		0.41						0.77
γ-Terpinene	1054	99-85-4		t	0.17	0.25	0.04	0.13	t	0.05
Borneol	1165	507-70-0								
Terpinen-4-ol	1174	562-74-3				0.56				
Terpinolene	1086	586-62-9		0.34	1.57	3.48	0.38	2.06		
α-Pinene oxide	1099	1686-14-2		0.04	0.04		0.04			0.04
*trans-p*-Mentha-2,8-dien-1-ol	1119	7212-40-0					0.04			
α-Campholenal	1122	91819-58-8		t						
*cis*-*p*-Mentha-2,8-dien-1-ol	1133	22771-44-4							0.05	
*iso*-3-Thujanol	1134	7712-79-0								0.10
*trans*-Pinocarveol	1135	517-61-5		0.05	0.06		0.03		0.21	0.09
*cis*-Verbenol	1137	1845-30-3		t						
*trans*-Verbenol	1140	1820-09-3		0.07			t		0.13	0.16
Camphor	1141	76-22-2								0.03
*trans*-Pinocamphone	1158	547-60-4		t	0.03				0.16	t
Pinocarvone	1160	16812-40-1		t						0.12
Borneol	1165	507-70-0		t	t		0.04		0.04	0.08
*cis*-Pinocamphone	1172	15318-88-0							0.03	0.08
Terpinen-4-ol	1174	562-74-3		0.06	0.14		0.07	0.15		0.12
α-Terpineol	1186	98-55-5		0.06			0.24		0.13	0.07
neo-dihydro Carveol	1193	18675-34-8			0.06					
Myrtenol	1194	515-00-4		0.04					0.17	t
Myrtenal	1195	18486-69-6							0.18	t
Verbenone	1204	80-57-9		t			t			t
Thymol methyl ether ili	1232	1076-56-8		0.04	0.20					t
Carvacrol methyl ether	1241	6379-73-3		t						
Linalool acetate	1254	115-95-7		0.03						0.08
Bornyl acetate	1287	76-49-3	4.39	0.55	0.18	3.93	0.64	0.17	0.20	1.98
*trans*- Sabinyl acetate (IPP vs. Acetyl)	1289	53833-85-5		t						
2-Undecanone	1293	112-12-9								0.09
Dihydro carveol acetate	1306	20777-49-5		t						
(*Z*)-Pinocarvyl acetate	1311	73366-18-4		0.04			t			
Myrtenyl acetate	1324	1079-01-2		0.06			t			
δ-Elemene	1335	20307-84-0		0.18			t			0.04
Verbanol acetate	1340	73366-09-3				0.07				
Terpinen-4-ol acetat	1343	4821-04-9.				0.53				0.27
α–Cubebene	1345	17699-14-8	0.63	t	t		0.10	0.21		t
α–Terpinyl acetate ili Neoiso–dihydrocarveol acetate	1346 1356	80-26-2 20777-49-5	0.27	1.02	0.12	2.41	t		0.06	0.81
α-Longipinene	1350	5989-08-2							0.20	t
Longicyclene	1371	1137-12-8							0.07	
α-Ylangene	1373	14912-44-8		0.03	t				t	
α–Copaene	1374	3856–25–5	0.22	0.11	0.41	0.09	0.28	2.27	0.05	0.17
*trans*-Myrtanol acetate	1385	90934-53-5				0.24				
β–Cubebene	1387	13744-15-5	0.07	0.17	0.20		t			0.27
β-Bourbonene	1387	5208-59-3		0.12						
β-Elemene	1389	515-13-9		0.05	0.32	2.49	0.06	0.64		1.72
Sativene	1390	6813-05-4						t	t	
β-Longipinene	1400	41432-70-6							t	
Longifolene	1407	475-20-7			0.09		0.05		1.26	
β-Funebrene	1413	50894-66-1			t					
(*E*)– Caryophyllene	1417	87-44-5	14.00	8.50	21.07	5.94	11.70	24.69	9.13	4.81
β-Copaene	1430	18252-44-3		0.27	0.27	0.09				0.10
Aromadendrene	1433	489-39-4								0.14
(*Z*)-β-Farnesene	1440	18794-84-8					0.06			
6,9-Guaiadiene	1442	36577-33-0				0.05	0.16			t
*cis*-Muurola-3,5-diene	1448	157374-44-2		0.08						
Spirolepechinene α-Himachalene	1449	246243-00-5 3853-83-6		t	0.08					
*trans*-Muurola-3,5-diene	1451	189165-77-3			0.07	0.10				
α– Humulene	1452	6753-98-6	2.35	1.53	3.70	1.07	2.01	3.85	1.56	0.85
(*E*)-β-Farnesene	1454	18794-84-8		0.10	0.21	0.35	t	0.12	t	t
Sesquisabinene	1457	58319-04-3		t				0.08		0.12
Alloaromadendrene	1458	25246-27-9		t						
*cis*-Cadina-1(6),4-diene	1461	000-00-0				0.44	t			
*cis*-Muurola-4(14),5-dien	1465	157477-72-0								0.12
Dauca-5,8-diene	1471	142928-08-3					t		t	
*trans*-Cadina-1(6),4-dien	1475	20085-11-4						0.07		t
α–Murrolene	1478	483-75-0	0.10	t			t		t	0.60
Amorpha-4,7(11)-dien-8-one	1479	000-00-0						0.10		
Germacrene D	1484	23986-74-5	1.39	16.34	16.30	5.50	0.20	1.51	0.07	3.80
Phenethyl 2-methylbutanoate	1486	24817-51-4		0.30				1.7		
β-Selinene	1489	17066-67-0								0.56
Phenyl ethyl 3-methyl-butanoate	1490	140-26-1		0.40				2.3		
*trans*–Muurola–4(14),5–diene	1493	54324-03-7	t		t	0.14	0.09			t
γ–Amorphene	1495	6980-46-7	0.24		t					
Valencene	1496	4630-07-3				0.14				
α–Muurolene	1500	31983-22-9	0.34	0.04	0.62	3.47	0.20		0.06	
Bicyclogermacrene	1500	67650-90-2			0.18	1.04				3.19
β–Bisabolene	1505	495-61-4					t	0.69		
Germacrene A	1508	28387-44-2		t	0.09	t				
δ- Amorphene	1511	189165-79-5		t						
γ–Cadinene	1513	39029-41-9	0.63	0.45	0.37	0.29	0.16			2.16
Cubebol	1514	23445-02-5						0.19		t
*cis*-Dihydroagarofuran	1519	150652-94-1		t						
Isobornyl isovalerate	1521	7779-73-9			t				0.06	
δ–Cadinene	1522	483-76-1	0.36	1.13	0.69	3.70	0.45	1.02	0.05	4.12
Isobornyl 2-methyl butanoate	1523	94200-10-9							0.06	
Zonarene	1528	41929-05-9							0.04	
α-Muurolene	1530	31983-22-9								t
(*Z*)-Nerolidol	1531	142-50-7							0.04	
*trans*-Cadina-1,4-diene	1533	38758-02-0		t	t	0.07	t		t	0.11
α- Cadinene	1537	24406-05-1		0.03	t	0.13				0.23
Germacrene A	1538	28387-44-2								t
α-Calacorene	1544	21391-99-1		t						t
*trans*-α-Bisabolene	1545	000-00-0			0.04					
Hedycaryol	1546	21657-90-9					0.12		t	
(*E*)-Veltonal	1555	58102-02-6		t						t
*trans*-Dauca-4(11),7-dien	1556	000-00-0		t						
*cis*-Muurol-5-en-4α-ol	1559	157374-45-3								0.05
*cis*-Muurol-5-en-4β-ol	1561	000-00-0								0.11
β –Calacorene	1564	50277-34-4		0.08						0.04
Longipinanol	1567	39703-23-6			0.06					
Germacrene D–4–ol	1574	74841–87–5	0.17	0.04	0.08	1.09				1.51
Spathulenol	1577	6750-60-3				1.39				
Caryophyllene oxide	1582	1139-30-6	0.55	0.42	0.78	0.67	0.80	1.00	0.66	
Germacrene D	1584	23986-74-5								
Thujopsan-2α-ol	1586	000-00-0			0.12					
Salvial-4(14)-en-1-one	1594	73809-82-2		t						
1,7,7-trimethyl acetate bicyclo[2.2.1]heptan-2-ol	1595	92618-89-8							t	
Longiborneol	1599	465-24-7							t	
Guaiol	1600	489-86-1		0.03			0.25	0.07	t	
Humulene oxide II	1608	19888-34-7		0.08	0.17	0.19	0.11	0.15	0.09	
1,10-diepi-Cubenol	1618	73365-77-2		0.04	0.08	t		0.08		
10-epi-γ-Eudesmol	1622	15051-81-7					t		t	
1-epi-Cubenol	1627	19912-67-5			t	0.08	0.08		0.08	
γ-Eudesmol	1630	1209-71-8					0.08		t	
α-Acorenol	1632	28296-85-7					t		t	
*cis*-Cadin-4-en-7-ol	1635	217650-27-6		t						
epi-α-Cadinol	1638	5937-11-1						0.30	t	
*allo*-Aromadendrene epoxide	1639	85160-81-2		0.24	t					
τ–Muurolol (*epi-*α-Muurolol)	1640	19912–62–0	0.07	t	0.10	1.05			t	
Torreyol	1644	19435–97–3	0.14		0.11	0.65	t	0.25	t	
Cubenol	1645	21284-22-0					0.11		t	
β-Eudesmol	1649	473-15-4					t		t	
α–Cadinol	1652	481–34–5	0.09	0.21	0.07	1.72	0.25	0.55	t	
Allohimachalol	1661	1891-45-8			t					
Intermedeol	1665	6168-59-8		0.04						
Bulnesol	1670	2245-73-6							t	
(*Z*)-Nerolidyl acetate	1676	91050-14-5		0.07					t	
Germacra-4(15),5,10(14)-trien-1α-ol	1685	81968-62-9		t						
Amorpha-4,9-dien-2-ol	1700	394251-66-2			t					
(2*E*)-Tridecanol acetate	1703	193758-89-3			t					
ar-Curcumen-15-al	1712	000-00-0							t	
14-hydroxy-α-Humulene	1713	000-00-0		t						
Oplopanone	1739	1911-78-0				0.15				
(*Z*)-Nerolidyl isobutyrate	1783	74646-27-8		t						
8 -Cedren-13-ol acetate	1788	18819-41-0		t						
1-Octadecene	1789	112-88-9			t					
Abieta-6,13-dien	1880	5939-62-8							0.08	
Khusinol acetate	1823	78405-34-2		t						
*cis*-Thujopsenic acid	1863	546-53-2		t						
1-Hexadecanol	1874	36653-82-4			0.44					
Cubitene	1878	66723-19-1		t						
Pimara-8,15-diene	1895	55255-56-6							t	
Rosa-5,15-dien	1902	1686-67-5							t	
epi-Laurenene ili Isopimara-9(11),15-diene	1901 1905	110455-92-0 39702-28-8					t			
Totarene	1922	000-00-0		t					t	
Beyerene	1931	3564-54-3							t	
Cembrene	1937	1898-13-1	0.44	0.70	0.11	0.18	t	1.05		
5α-androst-7-ene	1940	54411-76-6			t					
[4aS-(4aα,4aβ,7β,10aβ)]- 7-ethenyl-1,2,3,4,4a,4b,5,6,7,8,10,10a-dodecahydro-4a,7-dimethyl-1-methylene phenanthrene	1942	26549-04-2							t	
(3*E*)–Cembrene A	1947	31570-39-5	0.35	0.38				0.65		
Pimaradiene	1948	1686-61-9			t				0.09	
Hexadecanoic acid	1959	57-10-3			0.26					
(3*Z*)-Cembrene A	1965	71213-92-8						0.35		
(3*Z*)–Cembrene A	1967	71213-92-8	0.12	0.13						
Sandaracopimara-8(14),15-diene	1968	1686-56-2			0.28		0.06		0.12	
19-nor-Abieta-8,11,13-triene	1969	1686-61-9			t		t		t	
Sclarene	1974	511-02-4					0.19		0.44	
1,7,7-Trimethyl-3-phenethylidenebicyclo[2.2.1]heptan-2-one	1978	464-48-2							0.23	
Manool oxide	1987	596–84–9	0.52	0.06	0.29	0.25	0.45	0.14		
(9Z)-Octadecenal	1995	2423-10-1			t					
13–epi–Dolabradiene	2000	134507-28-1	1.98	t						
Phyllocladene	2016	20070-61-5	0.30	t		0.17				
8β,13β-kaur-16-ene	2017	20070-61-5							2.00	
Sclarene	2018	511-02-4			0.65					
Abieta–8,12–dien	2022	122712-77-0	0.42		0.20		0.06		0.80	
Geranyl linalool	2034	1113-21-9			t					
Kaurene	2042	34424-57-2	0.04							
Isocembrol	2047 (2073)	25269-17-4		0.97			t	2.45		
Abietatriene	2055	19407–28–4	0.40	t	0.31		0.34		1.04	
13-epi-Manool	2059	1438-62-6							0.27	
Abietadiene	2087	35241-400-8	0.45	t	0.46		0.03		1.50	
(*11E,13Z*)–Labdadien–8–ol	2095	000-00-0	18.83							
5-(7a-Isopropenyl-4,5-dimethyl-octahydroinden-4-yl)-3-methyl-pent-2-en-1-ol	2141	000-00-0			2.67		t			
Abienol	2149	25578–83–0	0.14	t	0.08		0.27		t	
Abieta–8(14),13(5)–dien	2153	5119–12–7	0.60		0.37		0.10		0.89	
Pimaral	2177	472-39-39					0.05			
Sandaracopimarinal	2184	3855–14–9	0.42	t	0.08		0.22		0.48	
Sclareol	2200	515-03-7		t						
Abieta-7,13-dien-3-one-18-al	2214	000-00-0			5.23					
Pimara–7,15–dien–3–one	2227	7715–48–2	2.03							
Methyl abietate	2234	127-25-3		t						
Methyl dehydroabietate	2341	1235-74-1		t						
Pimara-7,15-dien-3-ol	2253	4752-56-1					0.28			
Larixol	2265	1438–66–0	0.41							
Dehydroabietal	2274	13601–88–2	0.83	t	1.20		0.50		2.30	
Methyl isopimarate	2297	1686-62-0					t			
4-epi Abietal	2298	000-00-0							0.33	
Isopimardien-3-one	2300	000-00-0							0.36	
Abieta-7,113-dien-3-one	2312	29461-25-4							4.83	
Abietal	2313	6704–50–3	0.27		0.17		0.26			
8,13-Abietadien-18-ol	2324	21414-53-9			0.07				0.12	
Methyl dehydroabietate	2341	1235-74-1			0.08		t			
4-epi-Abietol	2343	24563-94-8							1.25	
Methyl neoabietate	2443	3310-97–2	0.62	t						
Methyl abietate	2356	127-25-3			0.11					
Methyl abietate	2380	127-25-3							1.32	
Dehydroabietol	2389	3772-55-2			t		0.20			
*p*-Anisic acid, 2-adamantyl ester	2395	000-00-0		t						
Neoabietic acid, methyl ester	2397	3310-97-2							0.17	
Abietol	2401	666-84-2							t	
Methyl neoabietate	2443	3310-97-2			t		0.08			
Abietic acid	2457	66104-41-4							t	
all-*trans* Retinal	2466	116-31-4			t		t			
Neo-abietol	2468	640-42-6			t				0.20	
22-methyl-24-norcholan-16-one	2515	54498-41-8			t					
Total			98.32	99.96	99.18	89.71	99.75	99.66	99.84	91.78
Monoterpene hydrocarbons			44.14	64.24	37.44	45.80	77.86	50.80	66.18	62.84
Oxygenated monoterpenes			5.29	2.58	2.40	10.98	1.58	2.59	1.36	4.16
Sesquiterpene hydrocarbons			19.70	29.03	44.71	25.34	15.42	35.23	12.65	23.11
Oxygenated sesquiterpenes			1.02	1.17	1.57	6.99	1.80	2.40	0.83	1.67
Diterpenes			5.62	1.27	2.93	0.60	1.23	2.19	6.96	
Oxygenated diterpene			23.55	0.97	7.02		1.86	2.45	11.63	
Others				0.70	3.11			4.00	0.23	

t-trace.

**Table 2:** Antimicrobial activity—MICs of *Pinus* sp. EOs.

MIC (μg/mL)	PMN	PMC	PNN	PNC	PSN	PSC	PHN	PHC
*S. aureus* ATCC 6538	1000	>1000	>1000	>1000	800	100	150	150
*E. faecalis* ATCC 29212	1000	1000	800	1000	600	100	150	100
*K. rhizophila* ATCC 9341	500	500	600	800	800	1000	400	800
*B. subtilis* ATCC 6633	1000	>1000	>1000	>1000	>1000	>1000	400	400
*E. coli* ATCC 8739	400	400	600	100	500	150	200	150
*K. pneumoniae* NCIMB 9111	600	600	500	800	600	400	400	500
*S. typhimurium* ATCC 14028	800	800	800	600	800	600	600	200
*P. aeruginosa* ATCC 9027	>1000	>1000	>1000	>1000	>1000	1000	800	>1000
*A. baumannii* ATCC 19606	>1000	>1000	>1000	>1000	>1000	>1000	>1000	>1000
*C. albicans* ATCC 10231	600	400	400	400	500	150	100	100

**Table 3:** Effects of combination of *Pinus* sp. EO and gentamicin against different bacterial strains. FIC of EO = MIC of oil in combination with antibiotic/MIC of EO alone. FIC of antibiotic = MIC of antibiotic in combination with EO/MIC of antibiotic alone. FIC index = FIC of EO + FIC of antibiotic. FICI ≤ 0.5, synergistic (SY); 0.5 < FICI < 1, additive effect (AD); 1 < FICI < 4, indifferent (IN).

Bacterial Strain		*S. aureus* ATCC 6538	*E. coli* ATCC 8739	*K. pneumoniae* NCIMB 9111
MIC (FIC)	Gent	0.0625 (0.125)	2 (1)	0.25 (0.25)
	PSC	75 (0.25)	37.5	37.5 (0.0375)
FICI (Eff)		0.375 (SY)	>1 (IN)	0.2875 (SY)
MIC (FIC)	Gent	0.125 (0.25)	2 (1)	0.25 (0.25)
	PNC	37.5 (0.25)	37.5	75 (0.075)
FICI (Eff)		0.5 (SY)	>1 (IN)	0.325 (SY)
MIC (FIC)	Gent	0.125 (0.25)	1 (0.5)	0.25 (0.25)
	PHN	37.5 (<0.0625) or 37.5 (0.0375)	75 (<0.125) or 75 (0.075)	37.5 (0.0375)
FICI (Eff)		<0.5 (SY) or 0.2875 (SY)	<1 (AD) or 0.575 (AD)	0.2875 (SY)
MIC (FIC)	Gent	0.125 (0.25)	1 (0.5)2	0.25 (0.25)
	PHC	37.5 (0.0625)	300 (<0.5) or 300 (0.3)	37.5 (0.0375)
FICI (Eff)		0.3125 (SY)	<1 (AD) or 0.8 (AD)	0.2875 (SY)

## Data Availability

The authors confirm that the data supporting the findings of this study are available within the article.
